# Understanding Cervicogenic Headache

**DOI:** 10.5812/aapm.3904

**Published:** 2012-07-10

**Authors:** Nicholas H L Chua, Hans V Suijlekom, Oliver H Wilder-Smith, Kris C P Vissers

**Affiliations:** 1 Department of Anesthesiology, Intensive Care and Pain Medicine, Tan Tock Seng Hospital, Singapore; 2 Department of Anesthesiology, Pain and Palliative Medicine, Radboud University, Nijmegen Medical Center, Nijmegen, The Netherlands; 3 Department of Anesthesiology and Pain Management, Catharina Hospital, Eindhoven, The Netherlands

**Keywords:** Post-Traumatic Headache, Headache, Secondary, Trigeminal Nucleus, Spinal, Chronic Pain, Neck

## Abstract

The purported mechanism underlying the development and progression of cervicogenic headache (CEH) is the convergence of sensory inputs at the trigeminocervical nucleus. This mechanism explains the radiation of pain from the neck or the occipitonuchal area and its spread to the oculo-fronto-temporal region; it also explains the recurrent headaches caused by improper neck postures or external pressure to the structures in the neck and the occipital region. These neural connectivity mechanisms involving the trigeminal nucleus are also evident from the eyeblink reflex and findings of quantitative sensory testing (QST). Understanding the mechanisms underlying the development of CEH is important because it will not only provide a better treatment outcome but will also allow practitioners to appreciate the variability of symptomatic presentations in these patients.

The International Headache Society classifies cervicogenic headache (CEH) as a secondary headache that has its nociceptive source in the neck and is perceived in one or more areas of the head and/or face (1). This classification system is mainly based on scientific diagnosis that requires the cervical nociceptive source to be identified via a confirmatory diagnostic block and the headache to have resolved after treatment. In contrast, the diagnostic methods described by the Cervicogenic Headache International Study Group (CHISG) (2, 3) are more practical and involve identifying important clinical markers specific for this type of headache. According to the CHISG, the most characteristic aspects of CEH are:

1. unilateral and radiating pain that often starts in the neck or the occipitonuchal area and spreads to the oculo-fronto-temporal region
2. temporal pattern of pain that is often continuous but fluctuates in intensity
3. pain induced by improper neck postures or external pressure to the structures in the neck and the occipital region.

Neuralgias, such as the greater, lesser, or third occipital neuralgias, affect similar regions at the back of the head. In contrast to patients with CEH, those with neuralgia often use terms such as “stabbing,” “jabbing,” or “shooting” to describe the pain intensity. In addition, neuralgias do not typically present with associated facial or trigeminal-referred pain. The reason for this observation is that the purported mechanism underlying the development and progression of CEH is the convergence of sensory inputs at the trigeminocervical nucleus (3, 4). A connection between the trigeminal and cervical nerves was postulated in the late 1940s (5), but it was only in 1961 that Frederick Kerr hypothesized a pathogenetic model for headache stemming from the cervical region and the posterior fossa (6). The trigeminal spinal nucleus comprises a rostral subnucleus oralis, a middle subnucleus interpolaris and a caudal subnucleus caudalis (7). The pars caudalis of the spinal tract nucleus of the trigeminal nerve is continuous with the grey matter of the dorsal horns of the spinal cord (8). The spinal terminals of the small sensory fibers enter the cord from the lateral part of the entry zone and have collateral branches that may ascend or descend for up to 3 segments, in the Lissauer’s tract, before synapsing in the dorsal horn laminae (9, 10). Therefore, along with the 3 upper segments, the middle and lower part of the neck may also be involved in the development of CEH (11–13).

These changes in neural connectivity are also evident in the findings of neurophysiological tests. The eyeblink reflex (BR) is mediated via the afferent fibers to the trigeminal sensory nuclear complex and their central connections in the trigeminal nucleus. The R1 and R2 components of the BR are mediated via the tactile Aβ afferent fibers. The R3 components are mediated via the thinly myelinated Aδ fibers. Sand *et al.* (14) compared the BR in patients with CEH, tension-type headache, and migraine with that in the controls. The initial study showed that shorter R1 latencies were found on the symptomatic side than on the asymptomatic side in patients with CEH. In a later study, they reported that stimulation of the symptomatic side in patients with CEH showed a decrease in the R2 duration and the amplitude of the R2 component. These findings point to an associated brainstem hyperactivity (15, 16), possibly involving the ipsilateral trigeminal nucleus.

The findings of the quantitative sensory testing (QST) of trigeminal hypersensitivity were consistent with those of the above-mentioned neurophysiological study. La Touche *et al.* (17) have reported that, compared to the pain-free controls, patients with chronic neck pain showed sensitivity to bilateral mechanical pain over the face. In these patients, pressure hyperalgesia was found over both the masseters and temporalis muscles, but not over the tibialis anterior muscle (reference area). However, Chua *et al.* (18) have shown that, compared to chronic neck pain patients without CEH, those with CEH showed lateralization of pressure hyperalgesia accompanied by thermal hyperesthesia on the painful side of the face. Their suggestion of a rostral neuraxial spread of central sensitization, probably to the ipsilateral trigeminal spinal nucleus, is consistent with Kerr’s (6) hypothesis.

Understanding the mechanisms underlying the development of CEH is the first step toward providing these patients with a better treatment outcome. This understanding will help rationalize the proposed mechanistic approaches that target central sensitization, ablative therapies that focus on primary nociceptive sources, and physical therapies that help relieve pain in secondary areas.
